# Expression of PD‐L1 in medullary thyroid carcinoma—a new therapeutic target?

**DOI:** 10.1002/edm2.241

**Published:** 2021-02-20

**Authors:** Liliana Fonseca, Cláudia Freitas, Ana Caramelo, Catarina Eloy

**Affiliations:** ^1^ Endocrinology, Diabetes and Metabolism Department Centro Hospitalar e Universitário do Porto Porto Portugal; ^2^ Institute of Molecular Pathology and Immunology University of Porto – IPATIMUP Porto Portugal; ^3^ Faculty of Medicine University of Porto Porto Portugal

## Abstract

PD‐L1 expression in MTC is hot topic since, if it is demonstrated that PD‐L1 is highly expressed in this cancer, thus immunotherapy against checkpoint inhibitors could become an important therapeutic tool in MTC treatment. To answer this question, we evaluated PD‐L1 expression in MCT tumour tissues, using an anti‐PD‐L1 22C3 antibody and found a high expression in 6 of the 8 patients (75%). Similarly, two other recent studies reported a higher PD‐L1 expression. According to our results, MTC cells present a significative PD‐L1 expression, raising the hypothesis that immunotherapy, such as pembrolizumab, could have a role on MCT treatment. The authors believe this is a fundamental question and may impact the future of MTC treatment.

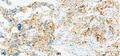

Nowadays, programmed death‐ligand 1 (PD‐L1) expression is being considered as a potential biomarker of response to anti‐PD‐1 or anti‐PD‐L1 agents in PD‐L1 positive tumours. Five PD‐1/PD‐L1 immunotherapies (atezolizumab, avelumab, durvalumab, nivolumab and pembrolizumab) have now been approved by the United States (US) Food and Drug Administration (FDA) and/or European Medicines Agency (EMA) for a variety of indications following the publication of clinical trials demonstrating their efficacy improving therapeutic response.^1^ So, if PD‐L1 expression in MTC is high, immunotherapy against checkpoint inhibitors could present itself as an important therapeutic tool, since medullary thyroid carcinoma (MTC) has a very high treatment refraction rate to conventional chemo and radiotherapy.^2^

Bongiovanni et al.´s described in their study a lower expression of PD‐L1 in MTC, namely of 6.25% (1/16) for tumoral and immune cells.^3^ On the contrary, two studies reported a higher PD‐L1 expression. Bi et al. described that PD‐L1 was expressed in 25.3% (22/87) and 21.8% (19/87) of tumour and immune cells, respectively,^4^ using the same antibody (SP263) and identical methods for scoring that Bongiovanni et al. In both studies^3^, ^4^ the threshold to consider a positive staining was a percentage of stained cells >1%. Moreover, Bi et al., also found a significant correlation between PD‐L1 expression and distant metastasis at surgery in MTC.^4^ Shi et al. reported, in the Chinese population, a higher PD‐L1 expression in tumour tissues of 14.4% (29/201) using PD‐L1 22C3 antibody. They demonstrated that PD‐L1 positivity was associated with clinicopathological features of aggressiveness and it was independently predictive of structural recurrence and biochemical recurrence/persistent disease. Furthermore, a higher rate of PD‐L1 expression has been found in patients with incurable recurrence.^5^ To our knowledge, only these three studies have previously evaluated PD‐L1 expression in MTC.^3^, ^4^, ^5^ Given the discrepancy in PD‐L1 expression prevalence between the three studies, we assessed MTC's PD‐L1 expression at our centre (Institute of Molecular Pathology and Immunology of the University of Porto ‐ IPATIMUP), in February 2020, analysing all cases evaluated from January 2011 to January 2020, with two different PD‐L1 clones.

Using a monoclonal mouse anti‐PD‐L1 clone, 22C3 (Dako, USA) and a rabbit monoclonal anti‐PD‐L1 SP142 (Ventana Medical Systems, Tucson, AZ) staining was performed on a BenchMark automated immunostainer (Ventana, Tucson, AZ, USA) using the OptiView DAB IHQ Detection Kit and Optiview Amplification Kit (Ventana Medical Systems, Tucson, AZ). We assessed PD‐L1 expression in both tumour cells and tumour‐infiltrating immune cells in the specimens (complete histological sections, not tissue microarray). For 22C3 we scored according to the guidelines of DAKO for urothelial carcinoma counting both expression in tumour cells and intratumoral immune cells; for SP142 we used the guidelines of ventana for urothelial carcinoma counting intratumoral immune cells only. The threshold to consider the staining as positive was a percentage of stained cells >1%. For positive cases, the percentage of stained cells was recorded.

For the 22C3 antibody, each specimen was regarded as PD‐L1‐positive if the combined positive score (CPS) was ≥1 (Figure 1). For the SP142 antibody, immune cell score was used.

**FIGURE 1:**
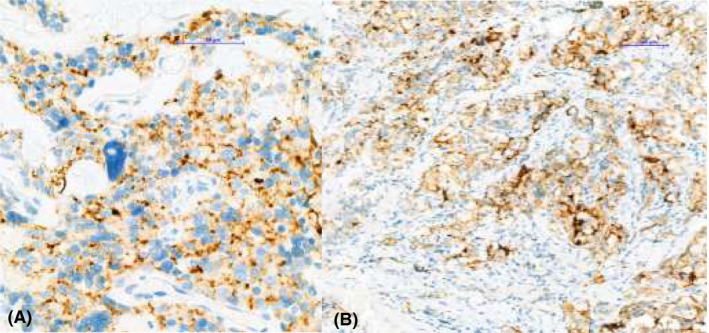
PD‐L1 (22C3 clone) expression in medullary thyroid carcinoma: A – case 1 (37×) and B – case 3(13×)

During the study period, eight cases of MTC, 4 females and 4 males, with a median age of 55 (min.‐max.: 37–74) years were evaluated. For tumour tissues, positive PD‐L1 expression was observed in 6 patients (75%) using anti‐PD‐L1 22C3. All samples scored negatively with anti‐PD‐L1 SP142 (Table 1).

**TABLE 1:** Clinicopathological data and PD‐L1 expression in malignant cells

Patient	Sex/age	Year; Type of surgery	Size, cm	pT	pN	PD‐L1 expression, CPS, 22C3	PD‐L1 expression, immune score, SP142
1	F/56	2012; Total thyroidectomy	0.8	1b	x	5	0
2^a^	F/37	2012; Lymph node metastasis recurrence	–	–	–	0	0
3	F/45	2017; Total thyroidectomy	0.4	1a	0	90	0
4	M/37	2018; Total thyroidectomy	1.8	1b	1	80	0
5	M/60	2018; Hemi thyroidectomy	0.6	1a	0	0	0
6	F/74	2019; Total thyroidectomy	1.7	1b	0	90	0
7^a^	M/65	2019; Lymph node metastasis recurrence	–	–	–	5	0
8	M/54	2020; Total thyroidectomy	2.3	2	x	5	0

Abbreviations: CPS, combined positive score; PD‐L1, programmed death‐ligand 1.

^a^ Co‐existence of lymph node metastasis at the time of surgery

According to our results, MTC cells presented a significative PD‐L1 expression with anti‐PD‐L1 22C3 use, raising the question of the potential efficacy of target therapeutics, such as Pembrolizumab, in selected MTC patient's treatment. Pembrolizumab is the most widely used drug and approved by Food and Drug Administration (FDA) to treat several different carcinomas, such as metastatic non‐squamous non‐small cell lung cancer, gastric or gastroesophageal junction adenocarcinoma, recurrent or metastatic head and neck squamous cell cancer with disease progression, refractory classical Hodgkin Lymphoma, locally advanced or metastatic urothelial carcinoma, unresectable or metastatic melanoma, and recurrent or metastatic cervical cancer.^6^

In our series, we detected a higher prevalence in PD‐L1 expression comparing to Bongiovanni et al.,^3^ probably because we used a different PD‐L1 antibody clone. In addition, Bongiovanni et al. looked at samples as old as 1996 which may have affected the expression of the PD‐L1, in our study the older tissue sample was 8 years old. In fact, we used an anti‐PD‐L1 22C3 to evaluate PD‐L1 expression and the specimen was regarded as ‘PD‐L1 positive’ if the CPS was ≥1, like in Shi et al..^5^ Although our PD‐L1 expression was higher than the latter, this could be due to our smaller sample size and different ethnicity of the enrolled patients.

Currently, there is no standard antibody use in MTC and the studies available in this area are few. We recognize our study small sample size as an important limitation. Furthermore, we lack data concerning patient follow‐up, since IPATIMUP is a molecular pathology and immunology centre of reference and thus, patient follow‐up is performed in other types of centres that may impact the future treatment of MTC. We have, however, found interesting results that raise several questions.

In conclusion, our study suggests that the use of the antibody 22C3 marker is associated with a higher PD‐1/PD‐L1 expression in MTC patients comparing with other antibodies. Do these results translate to more aggressiveness? Could pembrolizumab become a drug used in the treatment of MTC? Future studies with larger samples are needed to better understand the relationship between PD‐L1 expression and immune response.

## CONFLICT OF INTEREST

Liliana Fonseca declares that she has no conflict of interest. Cláudia Freitas declares that she has no conflict of interest. Ana Caramelo declares that she has no conflict of interest. Catarina Eloy declares that he has no conflict of interest.

## ETHICAL APPROVAL

All procedures performed in studies involving human participants were in accordance with the ethical standards of the institutional and/or national research committee and with the 1964 Helsinki declaration and its later amendments or comparable ethical standards.

## CONSENT FOR PUBLICATION

Liliana Fonseca assigns *Endocrinology*, *Diabetes & Metabolism* all rights of copyright.

## Data Availability

The datasets generated during and/or analysed during the current study are available from the corresponding author on reasonable request.
